# Validation of the “World Health Organization Disability Assessment Schedule for Children, WHODAS-Child” in Rwanda

**DOI:** 10.1371/journal.pone.0057725

**Published:** 2013-03-07

**Authors:** Pamela Scorza, Anne Stevenson, Glorisa Canino, Christine Mushashi, Fredrick Kanyanganzi, Morris Munyanah, Theresa Betancourt

**Affiliations:** 1 Harvard School of Public Health, Boston, Massachusetts, United States of America; 2 University of Puerto Rico, San Juan, Puerto Rico; 3 Partners in Health/Inshuti Mu Buzima, Rwinkwavu, Rwanda; University of Pennsylvania, United States of America

## Abstract

**Overview:**

The World Health Organization Disability Assessment Schedule for children (WHODAS-Child) is a disability assessment instrument based on the WHO's International Classification of Functioning, Disability and Health for children and youth. It is modified from the original adult version specifically for use with children. The aim of this study was to assess the WHODAS-Child structure and metric properties in a community sample of children with and without reported psychosocial problems in rural Rwanda.

**Methods:**

The WHODAS-Child was first translated into Kinyarwanda through a detailed committee translation process and back-translation. Cognitive interviewing was used to assess the comprehension of the translated items. Test-retest reliability was assessed in a group of 64 children. The translated WHODAS-Child was then administered to a final sample of 367 children in southern Kayonza district in rural southeastern Rwanda within a larger psychosocial assessment battery. The latent structure was assessed through confirmatory factor analysis. Reliability was evaluated in terms of internal consistency (Cronbach's alpha) and test-retest reliability (Pearson's correlation coefficient). Construct validity was explored by examining convergence between WHODAS-Child scores and mental disorder status, and divergence of WHODAS-Child scores with protective factors and prosocial behaviors. Concordance between parent and child scores was also assessed.

**Results:**

The six-factor structure of the WHODAS-Child was confirmed in a population sample of Rwandan children. Test-retest and inter-rater reliability were high (r = .83 and ICC = .88). WHODAS-Child scores were moderately positively correlated with presence of depression (r = .42, p<.001) and post-traumatic stress disorder (r = .31, p<.001) and moderately negatively correlated with prosocial behaviors (r = .47, p<.001). The Kinyarwanda version of the WHODAS-Child was found to be a reliable and acceptable self-report tool for assessment of functional impairment among children largely referred for psychosocial problems in the study district in rural Rwanda. Further research in low-resource settings and with more general populations is recommended.

## Introduction

The scope of health extends beyond the realm of disease to the wider domain of overall human functioning. Improvement in functional impairment is often a key criterion that society uses to evaluate the effectiveness of programs and treatments. Cross-cultural standardization of measurement of functioning has received considerable attention given its importance in evaluating global health programs as well as relating health status to economic development [1]. However, when assessing functional impairments in children and adolescents, vast contextual differences pose particular challenges. The role of children in household and community life differs across cultures, and resource constraints might impact the meaning of certain domains of functioning. For instance, many standard measures of daily functioning in children refer to school responsibilities and activities which may be less reliable questions for use in low-resource settings where some children may have limited access to school for reasons unrelated to functioning.

In order to reach a universally accepted conceptual framework to define and classify disability, the World Health Organization (WHO) developed the International Classification of Functioning, Disability, and Health (ICF). The ICF reflects a shift from biomedical and social models to a bio-psycho-social model, emphasizing the dynamic and bidirectional relations between a health condition and contextual factors (personal and environmental). In the ICF, disability is described as “a difficulty in functioning at the body, person, or societal levels, in one or more life domains, as experienced by an individual with a health condition in interaction with contextual factors” [2].

### Youth and Functioning

As demographic trends have resulted in a large percentage of the population in the younger age groups, the focus on youth in health and development has intensified. As clinicians and researchers used the ICF, they became more aware of its limitations for use with children and adolescents. More recently, the ICF has been modified to include foundational functional characteristics related to the developing child and the influence of environments surrounding the child. Derived from a linearization of the updated ICF, the International Classification of Functioning, Disability and Health Children and Youth version (ICF-CY) provides common terminology for identifying functional problems in children, including bodily functioning, activity limitations and participation restrictions. The ICF-CY is meant to provide a universal language for clinical, public health, and research applications to facilitate the documentation and measurement of health and disability in child and youth populations.

As part of the ongoing development of the ICF conceptual model, the World Health Organization Disability Assessment Schedule II (WHODAS-II) was created in1998 as a self-report tool that could be administered in both clinical and epidemiologic work. However, this tool was not designed for use in children. While clinician interview tools, such as the Children's Global Assessment Scale (CGAS) have shown validity for measuring functioning in children [3], a brief, standard scale that could be used to assess overall functioning in children was lacking. Recently, the WHODAS-II was modified for use with children, based on the ICF-CY (Canino et al- unpublished). The Child WHODAS-II was adapted for children from the adult WHODAS-II [4] by the Diagnostic and Statistical Manual of Mental Disorders (DSM) Version 5 Impairment/Disability workgroup (National Institute of Mental Health, 2005) of which one of the authors (GC) of this paper belongs. Prior to adapting the instrument, Dr. Darrel Regier from the American Psychiatric Association contacted Dr. Bedirhan Ustun from the WHO to ask permission to adapt the adult WHODAS for children. Dr. Ustun said that permission for this was not necessary and he encouraged the group to develop such an instrument. The adaptation process included making sure that the items could be well understood by children and their families, and that the items were consonant with the basic assumptions of child disability described in the ICF-CY [5]. In some occasions it was not possible to retain an item as presented in the WHODAS-II because it was not developmentally appropriate. For example, the item “How much of a problem did you have because of barriers or hindrances in the environment?” needed to be altered for comprehension by children. It was modified to read, “How much do you feel you were not getting invited to as many parties, play dates, or just hanging out, as you would like?” However, in the process of adaptation, the committee always considered the intent of the adult WHODAS-II item and tried to find a way of measuring the construct in a developmentally appropriate way.

The WHODAS-Child is currently undergoing field tests, and its suitability with children in a rural African population such as Rwanda has yet to be assessed. This current study analyzes the suitability and measurement properties of the WHODAS-Child in a population sample of youth in rural Rwanda, the majority of whom were referred for psychosocial problems. This analysis is situated within a larger study measuring mental disorders and protective processes in children in a district in southeastern Rwanda, southern Kayonza. The WHODAS-Child and other assessment tools in the battery will be used to evaluate a family-strengthening intervention that is being developed to prevent mental disorders in children in at-risk, HIV-affected families in this area.

Since the advent of version III of the DSM [6], the designation of a mental disorder has included disability or functional impairment in order for a disorder to be considered as present. A cluster of depressive symptoms does not meet DSM or ICD criteria for depressive disorder unless there is clinically significant impairment. Surveys that have devised ways to incorporate severity or level of impairment into the operational definition of “caseness” for mental disorders reduce rates of disorder by two- or threefold [7]. When translating mental disorder classification and diagnosis into different cultures, this component of disability becomes an important audit of the translation of the symptom clusters and disease classifications in the new context. As mental disorders are increasingly being recognized as one of the most important contributors to disease burden worldwide [8], [9], research and implementation focus is increasingly being devoted to their prevention and treatment. Because most mental disorders begin in childhood and adolescence [10], children are a target population for the prevention and treatment of mental disorders worldwide. Therefore, the absence of a standard measure that can be used in research to assess functioning and disability in children has major implications for a crucial area of health and development research- child mental health. This study examines the acceptability and metric properties of the WHODAS-Child for assessing functional impairment in children, the majority of whom have psychosocial problems, in rural Rwanda.

## Methods

### Ethics statement

Parental informed consent and child assent was obtained from all participants, either by written signature or by fingerprints, depending on literacy. Informed consent was confirmed by interviewers and was recorded in the smartphone form used for the interview. This consent protocol, as well as the larger study, was approved by the IRB of the Harvard School of Public Health and the Rwandan National Research Ethics Commission (RNEC). A referral system was in place such that if a child reported suicidal ideation or plans, the child was referred to the district hospital to see a psychologist. Given that no information on the validity of the WHODAS-Child was available, a similar referral system was not used for children scoring high on functional impairment.

### Sample

Data collection took place between March and December 2011 in southern Kayonza district, a region where our implementation partner, Partners in Health-Rwanda/*Inshuti Mu Buzima* (PIH/IMB), provides services. Participants were recruited from a catchment area of an estimated 157,270 residents. Those eligible for the study were Rwandan children and adolescents ages 10–17 and their caregivers. This age range was identified by PIH/IMB and the Rwandan Ministry of Health as a particularly underserved part of the population for mental health services. Exclusion criteria were: having lived in the region for less than a month, inability to speak Kinyarwanda fluently, and presence of severe cognitive impairment, as determined by study psychologists. Study recruitment aimed to identify participants exhibiting symptoms of locally defined mental health syndromes—including *agahinda kenshi (persistent sorrow)*, *kwiheba (severe hopelessness)*, *guhangayika (anxiety/depression)*, and *umushiha (persistent irritability/anger)—* as well as participants exhibiting none of these mental health syndromes. These syndromes were identified in prior qualitative research [11]. Table 1 presents a brief description of these syndromes and their approximate Diagnostic and Statistical Manual of Mental Disorders Text Revision (DSM-IV TR) correlates based on face validity in review by Rwandan psychologists and US-based research team members trained in DSM IV-TR diagnoses. *Agahinda kenshi* scale scores were strongly correlated with diagnosis of Major Depressive Episodes [12], and analyses of the correspondence between other syndromes and DSM diagnoses are in preparation. To identify children with and without these problems, study supervisors asked community health workers (CHWs), teachers, and health center staff to generate lists of children and adolescents ages 10–17 with each of these mental health syndromes, and a list of children with none. In Rwanda, CHWs are each assigned to track the health and wellbeing of approximately 50 families, making the CHW an excellent source of information on families residing in the villages in which the CHW works. Referral agents determined which children had each syndrome based on their knowledge of the children they knew well and their understanding of the syndrome cover term. Incentives given for participation in the study were books and pens worth approximately $1. The breakdown of children in the final sample by referral status is presented in Table 2.

**Table 1:** Local Mental Health Syndromes.

Name	Defining symptoms	Similar DSM-IV syndrome
*Guhangayika*	▪ State of constant worry or stress	Generalized anxiety disorder [300.02]
	▪ Person is never at ease	
	▪ Overthinking of problems without being able to find a solution	
	▪ Unwillingness to interact with others	
*Agahinda kenshi*	▪ State of persistent sadness or sorrow	Dysthymia [300.4]- Major depressive disorder [296.3]
	▪ Loneliness	
	▪ Unhappiness and low morale	
	▪ Crying	
*Kwiheba*	▪ Severe hopelessness	Severe Major depressive disorder [296.3]
	▪ Suicidal ideation	
	▪ Feeling that life is meaningless; pessimistic	
	▪ Uninterested in interpersonal interactions	
*Umushiha*	▪ Persistent irritability or anger	Severe Mood Disregulation [Proposed for DSM 5]
	▪ Grouchiness; rudeness	
	▪ Prone to quarreling	

**Table 2:** Demographics of participants N = 367.

Characteristic	N (%)
Sex	
Male	217 (59.1)
Female	150 (40.9)
School Status	
In School	278 (75.7)
Not in School	89 (24.3)
Age (years)	
10	40 (10.9)
11	45 (12.2)
12	51 (13.9)
13	53 (14.4)
14	59 (16.1)
15	40 (10.9)
16	29 (7.9)
17	50 (13.6)
Referral Status	
Agahinda Kenshi/Kwiheba *(depression like problems)*	58 (15.4)
Guhangayika *(worry/anxiety)*	94 (25.0)
Umushiha *(anger/irritability local syndrome term)*	50 (13.3)
Uburara *(conduct problems)*	51 (13.6)
healthy/free of mental disorders	123 (32.7)

Blind child clinical assessments and a battery of psychosocial assessments including the WHODAS-Child were administered to a total of N = 378 child and parent dyads in their homes. Eleven interviews were excluded from analysis due to withdrawals, leaving a sample of 367 (97.1%) for data analysis. Measures were administered to children and their caregivers separately, with the parent/caregiver reporting on the child. Data were collected by a study team of six Rwandan interviewers who were blind to the referral status of the child. Oversight was provided by local research coordinators, a field-based research manager and the study Principle Investigator, who hosted weekly supervision calls. All study staff were trained in research ethics and quantitative research techniques. Data were collected electronically using Samsung Galaxy GT 15503 smartphones running on an Android platform and uploaded to DataDyne's episurveyor.org website for data monitoring and downloading.

### Assessment instrument

The WHODAS-Child is a self-report assessment of difficulties in six domains: understanding and communicating, getting around (mobility), self-care (ability to attend to personal hygiene and safety), getting along with people, life activities (ability to carry out responsibilities at home, work and school), and participation in society (ability to engage in community, civil, and recreational activities). Due to literacy constraints, the 36-item Interviewer-Administered Version (youth report) and 36-item Interviewer-Administered Proxy Informant Version (parent/caregiver report) of the questionnaires were used to assess functioning in the target population.

#### Parent and Youth Versions

The parent and the youth versions begin with a global rating of overall health in the past 30 days, rated on a five-point scale (very good, good, moderate, bad, very bad). This is followed by 34 items divided across the different domains as follows: understanding and communicating - 6 items; getting around – 5 items; self-care – 4 items; getting along with people – 5 items; life activities – 4 items for non-school and 5 items for school; participation in society - 5 items. For each, the respondent considers the level of difficulty on a five-point scale (none, mild, moderate, severe, extreme/cannot do). At the end of the questionnaire the participant is asked to provide an overall rating of how much his/her difficulties interfered with the child's life (using the same five point scale), the number of days (of the last thirty) the difficulties were present, the child was unable to carry out usual activities or the child had to cut back on usual activities, how many days the child was late for school or absent from school. Children who were not enrolled in school were not asked these school-related questions.

#### Scoring

While a standard scoring method has not been defined by the WHO, for this analysis the WHODAS-Child scoring in this study was closely based on previous scoring of the WHODAS-II [4]. The only modification was to exclude the questions on school participation for those children who were not in school. This was done to avoid the problem of family resource limitations being confounded with functional impairment from physical or mental health conditions as the reason for limitations in school participation. While some children may be out of school because of physical or mental problems- for example, conduct disorder- our qualitative research indicated that school participation was more often determined by family resource constraints in this setting.

Aside from this modification, scoring followed a weighting system previously used with the adult version [4]. For the Understanding and Communicating, Getting Around, Self-Care, and Getting Along with Others domains, and Life Activities domains, the WHODAS-Child was scored by estimating the percent of maximum possible score observed for the items in that domain. The same was done for the two items asking about overall quality of health and impairment (h1 and h2). For impairments in usual activities, a weighted sum of activity limitation days in the prior month was estimated by adding together: (1) The number of days totally unable to carry out normal activities in the prior month (item H4); (2) One-half the number of days of reduced activities (item H5); (3) One-quarter the number of days difficulties were present (item H3); and (4) One-quarter the number of days the child was late for school (item H6). A global disability score was then estimated by averaging the scores of the Understanding and Communicating, Getting Around, Self-Care, Getting Along with Others, Life Activities, overall impairments, and activities limitation impairments sections. The global disability score had a potential range from 0–100. It is important to note that the Life Activities domain of the WHODAS-Child includes five questions about impairments in school activities and participation. Children who were not in school were not asked these five questions, and their score for that domain was the percent of maximum possible score in that domain excluding the five school-related questions.

#### Cross-cultural adaptation

When using self-report tools in a different language, a thorough translation and pre-testing process is recommended prior to assessing the reliability and validity of the tool [13], [14], [15]. The WHODAS was translated to the Rwandan local language, Kinyarwanda, according to standard protocols [16], [17], [18]. Two native speakers of the target language worked independently to forward-translate measures from the source language (English) to the target language (Kinyarwanda). These translators also made use of qualitative data collected in previous phases of the study describing symptoms of mental health problems in children and adolescents. In order to retain local rather than technical language, the local research team offered recommendations as needed. For example, the item “How much do you think that you are not getting invited to as many parties, play dates, or just hanging out, as you would like” had to be modified so that the social inclusion aspect of “play dates” was captured in a context where there are no “play dates” as such. The Kinyarwanda “*ni ku ruhe rugero utekereza ko udatumirwa nk'uko wabyifuzaga mubirori n'imikino binyuranya cyangwa aho uba uri kumwe n'abandi*” captures this social exclusion element of impairment by asking about not being included in games or social events.

An expert committee, composed of all three translators and a final bilingual adjudicator (who was knowledgeable of clinical psychological terms, a native speaker of the target language, and familiar with the study population), assembled to examine the instrument's cultural acceptability, resolve discrepancies among translations, and correct any problems with clarity, comprehension or language. This process synthesized the two translations to produce the measure in its final form. The resulting document was then back translated by an independent, bilingual reviewer. Once translated, a native English speaker compared the back translation with the original English to confirm parity. This rigorous translation process was completed to provide the research team with conceptually equivalent baseline versions of the standardized measure in English and Kinyarwanda [19]. Borrowing from graphics developed in Rwanda for prior research on functional impairments in adults [20], the research team used a visual display to represent the different response options of these Likert scale choices.

### Cognitive testing and reliability testing of measures

Cognitive testing of the measures took place in early 2011 with N = 25 children age 10–17. Interviews were conducted in the PIH/IMB catchment area in Kirehe district, a municipal region to next to southern Kayonza, to avoid overlap with the validation study sample. Respondents were asked all of the questions on the adapted WHODAS-Child scale in Kinyarwanda. After each question, following guidelines for cognitive testing of items [21], research assistants asked a set of structured questions in Kinyarwanda examining comprehension (respondent interprets the question), retrieval (respondent searches memory for relevant information), judgment (respondent evaluates/estimates response), and response (respondent provides information in the format requested) to understand what participants thought the question was asking and how each participant selected his/her response. A local research assistant wrote down the responses verbatim and also documented whether the respondent needed any part of the question repeated, had difficulty with the response options, or needed any clarification on the question. All responses were written down in a cognitive testing template and were then translated into English. The feedback was then reviewed as a group with the study team and the Principal Investigator. If the group found that respondents were struggling with a question or part of a question, it was discussed and altered as needed. Questions that were altered underwent another round of cognitive testing.

Reliability testing involved n = 34 children who were re-interviewed by the same interviewer 1–2 days after the initial interview to examine test-retest reliability, and n = 30 children were re-interviewed by a *different* interviewer 1–2 days after the initial interview, to assess inter-rater reliability.

### Analytical Strategy

Confirmatory factor analysis (CFA) was performed to assess the hypothesized six-domain structure of the WHODAS-Child, i.e. understanding and communicating, mobility, self-care, getting along with people, life activities, and participation in society. As WHODAS-Child responses are categorical variables, the factor analyses were based on polychoric correlations, and robust-weighted least squares estimators were used. With 36 items and 630 correlations, a six-factor CFA is over-identified, so model identification is not problematic. With a sample size of 367, the dataset met minimum sample size guidelines for CFA [22]. Given very few responses in the most severe category of Likert ratings, the last two response categories were collapsed into one category, reducing the number of parameters to be estimated.

Goodness-of-fit was measured by the Root Mean Square Error of Approximation (RMSEA, adequate if below .08), and the Comparative Fit Index (CFI) and Tucker Lewis Index (TLI), which are recommended to be over .95. These analyses were conducted with MPlus 6.0 [23] and missing values were considered missing at random. By default, Mplus uses likelihood estimation to handle missing data. This was deemed appropriate given low rates of missing data in this study (5.9%).

Distribution of WHODAS-Child global disability scores and separate domain scores were examined for the whole sample. Reliability was assessed in terms of internal consistency, inter-rater and test-retest reliability. The former was evaluated with Cronbach's alpha coefficients and the latter two by Pearson's correlation coefficient. Correlation between time 1 and time 2 scores at or above r = .7 was taken to indicate good reliability [24]. An intraclass correlation coefficient (ICC) was calculated to assess inter-rater reliability between different interviewers. This was to account for absolute and not only relative differences between reporters. Difference in WHODAS-Child scores by sex were assessed with a t-test, and differences by age were assessed by a one-way MANOVA.

#### Construct validity was assessed in two ways

1) Convergent validity with presence of mental disorders- major depressive episode, anxiety, post-traumatic stress disorder (PTSD), conduct disorder, and oppositional defiant disorder. These disorder statuses were based on clinician diagnosis assisted by a structured diagnostic interview. Two Rwandan psychologists administered the Mini International Neuropsychiatric Interview for Children and Adolescents (MINI KID) [25], a structured diagnostic interview for children based on DSM-IV and ICD-10 diagnoses. The local psychologists were trained in MINI KID administration by a senior psychiatrist and one of the creators of the MINI KID, Dr. Juris Janavs. Given that WHODAS scores were continuous and disorder classification was dichotomous, point biserial correlations were calculated between WHODAS-Child scores and disorder diagnosis.

2) Divergent validity with measures of prosocial behaviors and protective factors: Four measures of protective factors were included in the assessment battery: *kwizerana* (trust and togetherness in the family), *kwihangana* (perserverance), *kwigirira ikizere* (self-esteem/self-confidence), and *ubufasha abaturage batanga* (community support). These constructs were drawn from the results of a qualitative study in the district area in 2010 [26], and were measured by standard scales when possible and by scales created from qualitative data when no suitable standard scale existed. A scale of prosocial behaviors was also constructed, following the method outlined by Bolton for measuring functioning [27]. In brief, free list interviews asked local people about tasks children do that are helpful to others and good for the community. A community-specific prosocial questionnaire was then constructed based on the most commonly listed prosocial behaviors. The internal consistency of the resulting scale was high (alpha = .90), and scores were negatively correlated with diagnosis of major depressive episodes [12]. Scale scores for the protective factors and prosocial behaviors were continuous, and Pearson's correlation coefficients were calculated for correlations between WHODAS-Child scores and protective factor and prosocial behavior scale scores.

Agreement between child and parent reports of functioning was assessed in terms of difference in mean score, as well as correlation measured by Pearson's correlation coefficient. All statistical analyses aside from the CFA were performed using STATA version 11 [28].

## Results

Demographic characteristics of the participants (N = 367) as well as their referral status are shown in table 2. Cronbach's alpha for all items in the WHODAS-Child was .84. A six-factor model with the factors comprising the six domains of the WHODAS provides evidence that the structure of the WHODAS-Child was reproduced in this population. The value of the Root Mean Square Error of Approximation (RMSEA), .048 (90% CI .044–.053) indicated good fit for the six-factor model [29], and the CFI, .93, also indicating acceptable model fit [30]. Only two items, both in the “participation in society” domain, had factor loadings below .8. The lowest, .57, was the item “In the last 30 days, how much do your parents or other family members spend on your health condition or problems that you may have?” It is possible that this item does not sufficiently tap the underlying domain of limits in participation in society, even though it closely resembles the item in the *participation in society* domain of the WHODAS II, “How much of a problem did your family have because of your health problems?” Another possibility is that children are not able to accurately report this information. In our sample children reported that their family spent more time on their condition than the parents themselves reported- 45.6% of children reported that their family spent “a lot” on their health condition, while only 12.3% of parents reported spending “a lot” on the child's health problems. The other item, with a loading of .70 was the previously discussed social inclusion item which had to be modified from the culturally inappropriate terms in the English version- “In the last 30 days, how much do you think that you do not get invited to as many parties, play dates, or just hanging out, as you would like?” The Kinyarwanda adaptation of the item translates as “In the last 30 days, how much do you think that you are not invited to games or social events where you could be with others?”

**Figure 1:**
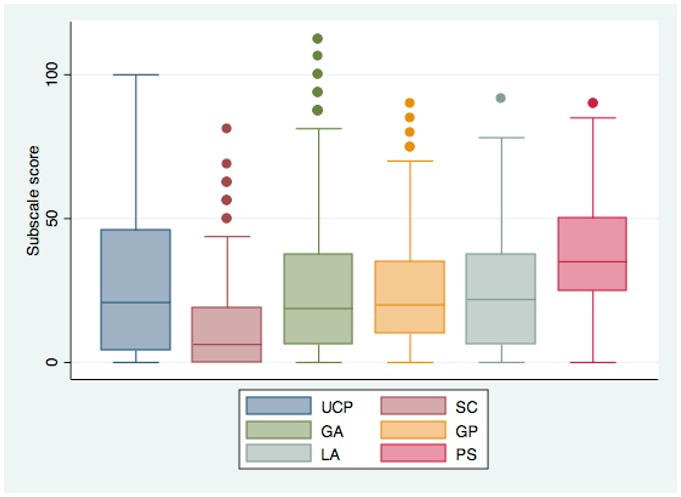
Distribution of WHODAS-Child Subscale Scores. Legend: Subscales, from left to right: Understanding and Communicating, Self-Care, Getting Around, Getting Along with People, Life Activities, Participation in Society. Bars represent 5^th^ and 95^th^ percentiles.

The distribution of WHODAS-Child scores was approximately normal, with a mean of 25.0 and a standard deviation of 14.57. The minimum WHODAS-Child score in this sample was 0, and the maximum was 81.3. Figure 1 shows distributions for subscale scores. Test re-test reliability of the WHODAS-Child was r = .83, and inter-rater reliability was ICC = .88. Mean WHODAS-Child scores were not significantly different for boys and girls, or by age, regardless of whether the parent or child was reporting (p = .32 for sex and p = .60 for age). WHODAS-Child scores were moderately positively correlated with major depressive episode and post-traumatic stress disorder diagnosis and positively correlated, but to a lesser extent, with anxiety and conduct disorder diagnosis. WHODAS-Child scores were inversely correlated with prosocial behaviors and protective factors. Tables 3 and 4 display these correlations. Mean WHODAS-Child scores were significantly higher for those youth who had a diagnosed mental disorder, compared to children with no disorder (29.4 versus 19.8, p<.001). Correlation between parent and child-report scores was r = .32. The mean difference in child and parent report scores was 3.8, with parents reporting significantly less impairment than children (p = .017). WHODAS scores based on parent reports were significantly less strongly correlated with diagnosis of major depressive episodes (r = .23 using parent reports versus p = .42 using child and adolescent reports, p = .004).

**Table 3:** Correlations between syndromes and WHODAS-Child scores.

Syndrome	Correlation	p-value
Major Depressive Episode	.*42*	<.001
Post-traumatic Stress Disorder	.*31*	<.001
Anxiety	.*18*	<.001
Conduct Disorder	.*18*	<.001

**Table 4:** Correlations between protective factors and WHODAS-Child scores.

Protective Factor	Correlation	p-value
Prosocial behaviors	−.47	<.001
Uburere Bwiza *Good parenting*	−.31	<.001
Kwizerana *Family togetherness*	−.34	<.001
Kwihangana *Perseverance*	−.34	<.001
Ubufasha *Community support*	−.17	<.001

## Discussion

This study examines the structure and psychometric properties of the WHODAS-Child, a recently developed version of the WHODAS-II for children and youth, in a sample of Rwandan children ages 10–17 who were largely referred for further screening of mental disorders. The six-factor structure of the WHODAS-Child was reproduced in this population, supporting the validity of the ICF-CY-derived domains in a sub-sample of largely referred children and adolescents in a rural African setting. While the responsibilities of daily life for children in rural areas may differ substantially from children in settings where the WHODAS was developed, the domains appear to be flexible enough to encompass important aspects of functioning in a markedly different environment. The relative importance of specific domains, however, as well as the likelihood for youth to experience difficulties in certain domains, may be different in this population than in other youth populations.

The domains of functional impairment that were most strongly correlated with all mental disorders in this sample were the two interpersonal domains- Understanding and Communicating, and Getting Along with People, and the domain Getting Around. Difficulties in Life Activities and Self-care might only be seen at more severe levels of mental disorder in this setting, given the greater consequences of inability to perform these tasks in a context where children are often more responsible for household livelihoods and personal care than their counterparts in more urban and high resource settings [31]. If this is the case, and these interpersonal domains are the first to be affected in populations of rural, African youth, the significance of difficulties in these domains should perhaps be given greater priority. Childhood and adolescence is a critical phase of the life cycle for establishing identity and independence which are manifest in the development of interpersonal and communication skills. These skills are also highly important for future education and productive participation in work and community life. Because such domains of functioning have a large impact on the future growth of human capital, in assessing functional impairments among children and adolescents in low resource settings, these aspects of functioning might be considered with greater weight. This is a particularly salient consideration in areas where youth comprise a relatively large percentage of the population and therefore have the potential to accelerate the country's development, a potential that has been called the “demographic dividend” [32]. Such consideration of interpersonal functioning is also consistent with current interest by many behavioral economists in what are referred to as “non cognitive skills” [33].

An important feature of this study was the elicitation of child self-reports via the WHODAS-Child as well as caregiver reports of functional impairment in the child. Analyses of differences in parent and child reporting of health, functioning, abilities, and emotions have found wide variations in the correlations between informant scores [34], and those differences are thought to be related to a number of factors, including the specific domains being measured, health and psychosocial well-being of the parent and of the child, and age. We found that parents report lower levels of child functional impairment on average, and that child reports correlate significantly more highly with depression. This is in contrast to findings that parents generally report more child symptoms than young people report themselves [35]. Parents in this context may be unaware of the extent of impairment that exists in day to day functioning among child and adolescent members of their household, or they may underreport functional impairment in their children for social acceptability reasons. This suggests that parent reports should not be used as a substitute for youth self-reports in the assessment of functional impairment in this context among this age group.

Study limitations and generalizability considerations must also be noted. In this study, level of functional impairment was not assessed with a clinician-administered interview but rather was gained via child and adolescent self-report and caregiver report. An additional thorough assessment via clinician interview could have provided a gold standard assessment of impairment, given that the significance of functional impairment has largely been an issue of clinical judgment [36]. However, the positive correlations with depression and anxiety, diagnosed by clinicians with fully structured diagnostic instruments, and the negative correlations with prosocial behaviors deemed important locally, increase our confidence in the validity of the WHODAS-Child in this setting among our study population. Still, a clinician assessment of functional impairment would be useful to determine a cut point or several cut points for levels of meaningful functional impairment to be used in future research and program evaluation.

Another consideration in interpreting and generalizing these results is the nature of this sample. Approximately 67% of children in the sample in this analysis were referred by local health workers and community leaders as being affected by one or more mental health problems. The structure and functioning of the tool might, therefore, be slightly different in a less impaired, non-referred population. The fact that none of the youth in the study were receiving formal mental health services at the time they were recruited into the study might suggest that the sample consisted of relatively mildly disordered youth. However, the lack of services for mental disorders for youth in the area, as well as the lack of referral services, may mean that even youth with moderate to severe problems would not be receiving formal services. Still, the most extreme end of the functional impairment scale- for example, institutionalized children- was therefore not represented in this sample, and a range of levels of impairment was represented, including 33% of the sample who were referred as mentally healthy children. However, the fact that this sample was designed and selected such that two thirds of the sample had mental health problems makes the generalizability of these results to more general populations of youth in sub-Saharan African settings inadvisable. Given that the WHODAS-Child is meant to be a measure of functional impairment due to a range of health problems, including physical health problems, additional studies should examine the functioning of the WHODAS-Child in more general populations and populations with other types of conditions causing impairment.

Another limitation lies in the lack of a standard scoring system for the WHODAS-Child. For this analysis, the scoring process used in the adult WHODAS version in the World Mental Health Surveys was used. Scored accordingly, the WHODAS-Child showed promising convergent and divergent validity. Still, alternate scoring methods could be explored, and a standard scoring procedure is in the process of being developed following psychometric analyses of WHODAS-Child data in the United States.

As recognition of the importance of youth in health and global economic development increase, it will be of even greater importance that programs aimed at improving the lives of youth, including health and mental health programs, measure the impact of the program on functioning in children, adolescents, and youth. Such steps require that we have a better understanding on how tools to measure functional impairment perform in different populations, and more fundamentally, what this tells us about the importance of particular domains of youth functioning. This analysis supports the cross-cultural acceptability, reliability, and validity of the WHODAS-Child in a sample of rural Rwandan youth largely referred for mental health disorders. Further theoretical and empirical research should explore the functioning of the WHODAS-Child in more general populations as well as the relative importance of the particular domains of functioning assessed in the WHODAS-Child, so that evaluations of health and development programs can focus on domains with critical impact on youth development.

## References

[1] Murray CJ (2002) Summary measures of population health: concepts, ethics, measurement and applications: WHO.

[2] Ingstad B WS, editor (1995) Disability and Culture. Berkeley: University of California Press.

[3] Shaffer D, Gould MS, Brasic J, Ambrosini P, Fisher P, et al (1983) A children's global assessment scale (CGAS). Archives of General Psychiatry 40: 1228. 6639293 10.1001/archpsyc.1983.01790100074010

[4] Von Korff M, Crane PK, Alonso J, Vilagut G, Angermeyer MC, et al (2008) Modified WHODAS-II provides valid measure of global disability but filter items increased skewness. J Clin Epidemiol 61: 1132–1143 Epub 2008 Jul 1110. 18619808 10.1016/j.jclinepi.2007.12.009 PMC3277915

[5] WHO (2007) International Classification of Functioning, Disability and Health; Children and Youth Version. Geneva: World Health Organization.

[6] AmericanPsychiatricAssociation (1980) Diagnostic and statistical manual of mental disorders.

[7] Winters NC CB, Myers KM (2005) Ten-Year Review of Rating Scales, VII: Scales Assessing Functional Impairment. Journal of the American Academy of Child and Adolescent Psychiatry 44: 309–338. 15782079 10.1097/01.chi.0000153230.57344.cd

[8] Ustun TB, Ayuso-Mateos JL, Chatterji S, Mathers C, Murray CJ (2004) Global burden of depressive disorders in the year 2000. Br J Psychiatry 184: 386–392. 15123501 10.1192/bjp.184.5.386

[9] WHO (2008) mhGAP Scaling up care for mental, neurological, and substance use disorders. World Health Organization. 26290926

[10] Patel V FA, Hetrick S, McGorry P (2007) Mental health of young people: a global public-health challenge. Lancet 369: 1302–1313. 17434406 10.1016/S0140-6736(07)60368-7

[11] Betancourt TS, Rubin-Smith JE, Beardslee WR, Stulac SN, Fayida I, et al (2011) Understanding locally, culturally, and contextually relevant mental health problems among Rwandan children and adolescents affected by HIV/AIDS. AIDS Care 24: 1–12. 21271393 10.1080/09540121.2010.516333 PMC3057405

[12] Betancourt T, Scorza P, Meyers-Ohki S, Mushashi C, Kayiteshonga Y, et al (2012) Validating the center for epidemiological studies depression scale for children in rwanda. J Am Acad Child Adolesc Psychiatry 51: 1284–1292. 23200285 10.1016/j.jaac.2012.09.003 PMC5730330

[13] Matias-Carrelo LECL, Negron G, Canino G, Aguilar-Gaxiola S, Hoppe S (2003) The Spanish Translation and Cultural Adaptation of Five Mental Health Outcome Measures. Culture, Medicine and Psychiatry 27: 291–313. 10.1023/a:1025399115023 14510096

[14] Kohrt BA, Jordans MJ, Tol WA, Luitel NP, Maharjan SM, et al (2011) Validation of cross-cultural child mental health and psychosocial research instruments: adapting the Depression Self-Rating Scale and Child PTSD Symptom Scale in Nepal. BMC Psychiatry 11: 127. 21816045 10.1186/1471-244X-11-127 PMC3162495

[15] Prince M (2008) Measurement validity in cross-cultural comparative research. Epidemiol Psichiatr Soc 17: 211–220. 18924560 10.1017/s1121189x00001305

[16] Benjet C (2010) Childhood adversities of populations living in low-income countries: prevalence, characteristics, and mental health consequences. Current Opinion in Psychiatry 23: 356–362. 20520546 10.1097/YCO.0b013e32833ad79b

[17] Brislin RW (1970) Back-translation for cross-cultural research. Journal of Cross-Cultural Psychology 1: 158–216.

[18] McDermott BM, Lee EM, Judd M, Gibbon P (2005) Posttraumatic Stress Disorder and General Psychopathology in Children and Adolescents Following a Wildfire Disaster. Canadian Journal of Psychiatry 50: 137–143. 15830823 10.1177/070674370505000302

[19] Harkness J, Pennell BE, Schoua-Glusberg A (2004) Survey Questionnaire Translation and Assessment. In: Presser S, Rothgeb JM, Couper MP, Lessler JT, Martin E et al.., editors. Methods for Testing and Evaluating Survey Questionnaires. Hoboken, NJ: John Wiley and Sons.

[20] Bolton P, Neugebauer R, Ndogoni L (2002) Prevalence of depression in rural Rwanda based on symptom and functional criteria. J Nerv Ment Dis 190: 631–637. 12357098 10.1097/00005053-200209000-00009

[21] Lee J (2012) Conducting cognitive interviews in cross-national settings. Assessment 10.1177/1073191112436671 22327207

[22] MacCallum RC WK, Zhang S, Hong S (1999) Sample size in factor analysis. Psychological Methods 4: 84–99.

[23] Muthen LKMB (1998–2010) Mplus User's Guide. Sixth Edition. Los Angeles, CA: Muthen & Muthen.

[24] Murphy KRDC (1996) Psychological Testing: Principles and applications. New Jersey: Prentice Hall International, Inc.

[25] Sheehan D, Shytle D, Milo K, Janavs J, Lecrubier Y (2009) MINI International Neuropsychiatric Interview for Children and Adolescents, English Version 6.0.

[26] Betancourt TS, Meyers-Ohki S, Stulac SN, Barrera AE, Mushashi C, et al (2011) Nothing can defeat combined hands (Abashize hamwe ntakibananira): protective processes and resilience in Rwandan children and families affected by HIV/AIDS. Soc Sci Med 73: 693–701. 21840634 10.1016/j.socscimed.2011.06.053 PMC3162991

[27] Bolton P, Tang AM (2002) An alternative approach to cross-cultural function assessment. Soc Psychiatry Psychiatr Epidemiol 37: 537–543. 12395144 10.1007/s00127-002-0580-5

[28] StataCorp (2009) Stata Statistical Software. 11 ed. College Station, TX: StataCorp LP.

[29] MacCallum RCBM, Sugawara HM (1996) Power Analysis and Determination of Sample Size for Covariance Structure Modeling. Psychological Methods 1: 130–149.

[30] Hu L BP (1999) Cutoff criteria for fit indexes in covariance structure analysis: Conventional criteria versus new alternatives. Structural Equation Modeling: A Multidisciplinary Journal 6: 1–55.

[31] Adolfsson M (2012) Applying the ICF-CY to identify children's everyday life situations: A step towards participation-focused code sets. International Journal of Social Welfare doi:10.1111/j.1468-2397.2012.00876.x.

[32] Bloom D CD, Fink G, Finlay J (2007) Realizing the demographic dividend: Is Africa any different? Manuscript prepared for the African Economic Research Consortium ed.

[33] Heckman J, Stixrud J, Urza S (2006) The Effects of Congitive and Noncognitive Abilities on Labor Market Outcomes and Social Behavior. Journal of Labor Economics 24: 411–482.

[34] Cremeens J, Eiser C, Blades M (2006) Factors influencing agreement between child self-report and parent proxy-reports on the Pediatric Quality of Life Inventory 4.0 (PedsQL) generic core scales. Health Qual Life Outcomes 4: 58. 16942613 10.1186/1477-7525-4-58 PMC1564004

[35] Kramer TL, Phillips SD, Hargis MB, Miller TL, Burns BJ, et al (2004) Disagreement between parent and adolescent reports of functional impairment. J Child Psychol Psychiatry 45: 248–259. 14982239 10.1111/j.1469-7610.2004.00217.x

[36] Bird H (1999) The assessment of functional impairment. In: Shaffer D LC, Richters JE, editor. Diagnostic ASsessment in Child and ADolescent Psychopathology. New York: Guilford. pp. 209–229.

